# The relative effects of non-pharmaceutical interventions on wave one Covid-19 mortality: natural experiment in 130 countries

**DOI:** 10.1186/s12889-022-13546-6

**Published:** 2022-06-03

**Authors:** Jonathan Stokes, Alex James Turner, Laura Anselmi, Marcello Morciano, Thomas Hone

**Affiliations:** 1 Health Organisation, Policy & Economics (HOPE), Centre for Primary Care & Health Services Research, University of Manchester, Manchester, England; 2 Research Centre for the Analysis of Public Policies (CAPP), University of Modena and Reggio Emilia, Modena, Italy; 3 Public Health Policy Evaluation Unit, School of Public Health, Imperial College London, London, England

**Keywords:** Health policy, Covid-19, Public health

## Abstract

**Background:**

Non-pharmaceutical interventions have been implemented around the world to control Covid-19 transmission. Their general effect on reducing virus transmission is proven, but they can also be negative to mental health and economies, and transmission behaviours can also change voluntarily, without mandated interventions. Their relative impact on Covid-19 attributed mortality, enabling policy selection for maximal benefit with minimal disruption, is not well established due to a lack of definitive methods.

**Methods:**

We examined variations in timing and strictness of nine non-pharmaceutical interventions implemented in 130 countries and recorded by the Oxford COVID-19 Government Response Tracker (OxCGRT): 1) School closing; 2) Workplace closing; 3) Cancelled public events; 4) Restrictions on gatherings; 5) Closing public transport; 6) Stay at home requirements (‘Lockdown’); 7) Restrictions on internal movement; 8) International travel controls; 9) Public information campaigns. We used two time periods in the first wave of Covid-19, chosen to limit reverse causality, and fixed country policies to those implemented: i) prior to first Covid-19 death (when policymakers could not possibly be reacting to deaths in their own country); and, ii) 14-days-post first Covid-19 death (when deaths were still low, so reactive policymaking still likely to be minimal). We then examined associations with daily deaths per million in each subsequent 24-day period, which could only be affected by the intervention period, using linear and non-linear multivariable regression models. This method, therefore, exploited the known biological lag between virus transmission (which is what the policies can affect) and mortality for statistical inference.

**Results:**

After adjusting, earlier and stricter school (− 1.23 daily deaths per million, 95% CI − 2.20 to − 0.27) and workplace closures (− 0.26, 95% CI − 0.46 to − 0.05) were associated with lower Covid-19 mortality rates. Other interventions were not significantly associated with differences in mortality rates across countries. Findings were robust across multiple statistical approaches.

**Conclusions:**

Focusing on ‘compulsory’, particularly school closing, not ‘voluntary’ reduction of social interactions with mandated interventions appears to have been the most effective strategy to mitigate early, wave one, Covid-19 mortality. Within ‘compulsory’ settings, such as schools and workplaces, less damaging interventions than closing might also be considered in future waves/epidemics.

**Supplementary Information:**

The online version contains supplementary material available at 10.1186/s12889-022-13546-6.

## Background

Non-pharmaceutical interventions were considered one of the few options for reducing the transmission of Covid-19 prior to vaccines, and so have played a critical role in the policy response [1, 2]. Countries around the world have introduced, at different times, to varying strictness levels, and in different combinations, a range of interventions including: public information campaigns, school and workplace closures, public event bans and restrictions on gatherings, public transport shutdowns, restrictions on internal movement (within countries), international travel controls, and stay-at-home (‘lockdown’) requirements [3]. All interventions aimed to reduce virus transmission, and related morbidity and mortality, by reducing social contacts. Each intervention has an obvious mechanism to achieve these social contact changes, but social behaviours can also change voluntarily without mandated policies. Their implementation can also negatively affect the economy and other health outcomes, including mental health and chronic conditions [4–7]. Assessing the relative effectiveness of concurrent interventions is essential to inform the design, including timeliness and strictness, of high-impact mitigation strategies to enable policy selection for maximal benefit with minimal disruption.

Public attention on effectiveness of Covid-19 measures has largely focused on different countries’ strategies, with certain countries singled out as exemplars based on unadjusted descriptive analysis [8]. However, early decisions on the timing and strictness of measures were driven by political and international factors (such as supply of tests and personal protective equipment) and informed by predictive epidemiological modelling. For example, a prominent report estimated over half a million deaths in the UK and 2 million deaths in the US before end of October 2020 without interventions, not accounting for any additional effects of health systems being potentially overwhelmed [9]. Particularly in early parts of the epidemic, the estimates from these predictive models were based on assumptions of the reproduction rate of the virus and ability of each intervention to alter this rate. Many subsequent studies have since used updated knowledge and prospective simulations to assess the potential impact of interventions [10–12].

However, evidence is still needed empirically on the effectiveness of non-pharmaceutical interventions on Covid-19 mortality, particularly comparing specific intervention effects. A number of studies have examined effects of interventions observed in previous epidemics [13, 14], nothing like the scale of response as compared to Covid-19. Others have examined the effects of single interventions for Covid-19, predominantly stay-at-home requirements, without controlling for concurrent interventions which also affect the same outcomes [15–17], potentially biasing results. Some examine efforts in single countries as a bundle of interventions without looking at relative effects of each [18–20], limited to the bundle of interventions implemented in that context at a single point of time. More recent studies have reported observational evidence on the infection rate from multiple countries of bundles of interventions introduced in tandem, or in quick succession [1, 2], but the methods used likewise do not allow relative effects to be estimated.

Finally, there are studies, like ours, which attempt to examine relative intervention effects using multiple cross-country counterfactuals. For example, Kontis et al. examined all-cause mortality in 21 industrialised countries using Bayesian models to group countries in terms of overall death toll [21]. Relative success of interventions was then inferred via qualitative comparative analysis based on these groupings to discuss possible policy mechanisms. Haug et al. instead drew on four statistical methods (case-control analysis for each intervention in turn; step function Lasso to select a reduced set of interventions that best describe the change in outcome; Random Forest regression; Transformer modelling) and interventions implemented across 56 countries and 24 of the United States (79 territories in total) to examine relative effects on the effective reproduction number [22]. Liu et al. also examined effects of interventions on the reproduction number, drawing on panel regression (linear fixed-effects model – relying on within-country variation over time) and 130 countries [23]. Results have been mixed.

A key limiting factor to all of the analyses above (and this one too) is that there are no current best practice methods in health policy research for analysing multiple interventions introduced simultaneously, as has been the case in response to Covid-19. This lack of definitive methods makes it essential to examine the question using a variety of different methodological approaches to expand the evidence base and to complement inherent weaknesses in any single method.

We aimed to add to this literature base by examining the relative association between strictness and timing of individual non-pharmaceutical interventions and Covid-19 mortality in wave one, adjusting for concurrent interventions. Results complement the current literature and together inform the understanding of the drivers of differences in mortality across countries and policy choice for future waves and epidemics.

## Materials and methods

We analysed observational data from 130 countries and exploited cross-country variation in policy implementation. We used a novel approach in the current literature, exploiting the known time lag between intervention implementation and mortality [24–26] to mitigate reverse causality bias and used multivariable regression models.

### Data

Our outcome of interest was Covid-19 attributed mortality rate. We obtained daily Covid-19 deaths for 130 countries from the European Centre for Disease Prevention and Control (ECDC) website (https://www.ecdc.europa.eu/en/covid-19/data-collection) on 1st June 2020. The ECDC compiles data from multiple sources, mostly sourced from individual country Ministries of Health or National Public Health Institutes. This does indicate that the precise definition of Covid-19 death varies somewhat across country (which we examine in robustness checks, below), and it does not distinguish between deaths directly from- or deaths with-Covid-19. Covid-19 mortality was measured as country daily deaths per 1 million population, with World Bank country population estimates from 2018 used as the denominator. Days in each country were indexed in time by first reported Covid-19 death to proxy for virus transmission rate and to make rates comparable over time.

Our variables of interest measured countries’ implementation, timing, and strictness of non-pharmaceutical interventions. We obtained data on daily national intervention stringency from the Oxford COVID-19 Government Response Tracker (OxCGRT) on 1st June 2020 [3]. Data are collected by over 1 hundred members of the OxCGRT team from publicly available sources including news reports and government briefings, using a standardised template. We examined nine non-pharmaceutical interventions which were categorised according to their strictness (see Table 1).

**Table 1:** Intervention strictness coding (adapted from [3])

Indicator	Strictness Coding
School closing	0 - No measures 1 - Recommend closing 2 - Require closing (only some levels or categories, eg just high school, or just public schools) 3 - Require closing all levels
Workplace closing	0 - No measures 1 - Recommend closing (or work from home) 2 - Require closing (or work from home) for some sectors or categories of workers 3 - Require closing (or work from home) all-but-essential workplaces (e.g. grocery stores, doctors)
Cancelled public events	0 - No measures 1 - Recommend cancelling 2 - Require cancelling
Restrictions on gatherings	0 - No restrictions 1 - Restrictions on very large gatherings (the limit is above 1000 people) 2 - Restrictions on gatherings between 100 and 1000 people 3 - Restrictions on gatherings between 10 and 100 people 4 - Restrictions on gatherings of less than 10 people
Closing public transport	0 - No measures 1 - Recommend closing (or significantly reduce volume/route/means of transport available) 2 - Require closing (or prohibit most citizens from using it)
Stay at home requirements	0 - No measures 1 - Recommend not leaving house 2 - Require not leaving house with exceptions for daily exercise, grocery shopping, and ‘essential’ trips 3 - Require not leaving house with minimal exceptions (e.g. allowed to leave only once every few days, or only one person can leave at a time, etc.)
Restrictions on internal movement	0 - No measures 1 - Recommend closing (or significantly reduce volume/route/means of transport) 2 - Require closing (or prohibit most people from using it)
International travel controls	0 - No measures 1 - Screening 2 - Quarantine arrivals from high-risk regions 3 - Ban on high-risk regions 4 - Total border closure
Public information campaigns	0 -No COVID-19 public information campaign 1 - Public officials urging caution about COVID-19 2 - Coordinated public information campaign (e.g. across traditional and social media)

We also collated country-level co-variates (potential confounders), which might affect the outcome and the uptake of non-pharmaceutical interventions (see Table 2). These included available cross-country proxies for healthcare infrastructure and quality, demographics, and economic and governance indicators. We also controlled for a country’s testing and contact tracing policies in all regression models, where more stringent testing/tracing increases a country’s ability to attribute deaths to Covid-19. We used the most up-to-date available period comparable across countries (2018).

**Table 2:** Country co-variates data and sources

Measure	Reason for inclusion	Source
Population density (people per sq. km)	As an infectious disease, higher density of population is likely to aid spread	https://data.worldbank.org/indicator/en.pop.dnst
% Population aged 65+	Older persons more vulnerable to adverse effects of infection	https://data.worldbank.org/indicator/sp.pop.65up.to.zs
% Population male	Adverse effect of infection might vary by sex	https://data.worldbank.org/indicator/SP.POP.TOTL.MA.ZS
Life expectancy at birth (years)	To adjust for relative baseline health prior to pandemic	https://data.worldbank.org/indicator/SP.DYN.LE00.IN
Hospital beds (per 1000 people)	To adjust for relative hospital capacity prior to pandemic	https://data.worldbank.org/indicator/sh.med.beds.zs
Physicians (per 1000 people)	To adjust for relative workforce capacity prior to pandemic	https://data.worldbank.org/indicator/SH.MED.PHYS.ZS
GDP PPP (current international $)	Gross Domestic Product (at Purchasing Power Parity), comparable measure of country wealth and relative average living standard. To adjust for relative deprivation across countries	https://data.worldbank.org/indicator/NY.GDP.MKTP.PP.CD
Manufacturing, value added (%GDP)	Healthcare treatment responses to the pandemic required scale-up of various equipment (e.g. ventilators, testing equipment, and personal protective equipment), globally. Extent of manufacturing base might conceivably have changed how a country was able to respond to changes in demand internally	https://data.worldbank.org/indicator/nv.ind.manf.zs
Health expenditure (%GDP)	To adjust for relative importance given to health budgets prior to pandemic	https://data.worldbank.org/indicator/SH.XPD.CHEX.GD.ZS
International tourism, number of arrivals	The virus originated in Wuhan, China. Infectious disease spread from an external source (for all other countries) will conceivably vary by extent of international movement	https://data.worldbank.org/indicator/ST.INT.ARVL
Governance (Voice and Accountability)	Different governance structures might impact when and how policies were introduced, and how strictly they were adhered to. We use a measure which captures the extent to which a country’s citizens are able to select their government, freedom of expression, association and media, i.e. extent of democracy	https://info.worldbank.org/governance/wgi/Home/Documents#wgiDataCrossCtry
Region	The virus originated in the East Asia & Pacific region (Wuhan, China), so region might affect relative timing of virus arrival and any associated technological/virus evolution changes over time	https://data.worldbank.org/indicator/en.pop.dnst
Testing policy (h2)	With Covid-19 testing policy closely tied to attribution of Covid-19 deaths, testing policies will be inextricably linked to the outcome, i.e. more testing will offer more opportunity to attribute a death to Covid-19. We control for this difference by controlling for the extent of testing policy in a given country at a given time Coding: 0 - No testing policy; 1 - Only those who both (a) have symptoms AND (b) meet specific criteria (e.g. key workers, admitted to hospital, came into contact with a known case, returned from overseas); 2 - Testing of anyone showing COVID-19 symptoms; 3 - Open public testing (eg “drive through” testing available to asymptomatic people)	https://www.bsg.ox.ac.uk/research/research-projects/coronavirus-government-response-tracker
Contact tracing (h3)	As above, contact tracing is closely linked and reliant on testing policy/capacity. We additionally control for the extent of contact tracing policy in a given country at a given time Coding: 0 - No contact tracing; 1 - Limited contact tracing - not done for all cases; 2 - Comprehensive contact tracing – done for all cases.	https://www.bsg.ox.ac.uk/research/research-projects/coronavirus-government-response-tracker

### Analysis

We first highlighted descriptively variations in the implementation of non-pharmaceutical interventions across selected countries. We plotted both the stringency and timing of intervention implementation against daily number of deaths per 1,000,000 population over indexed time. We smoothed the series by plotting estimates from local linear regressions [27]. We also examined the unadjusted associations between interventions and Covid-19 mortality with scatterplots.

Our statistical analysis method exploited the known biological lag between virus transmission (which is what the non-pharmaceutical interventions can affect) and mortality. This approach was employed to address concerns of bias due to potential reverse causality caused by policymakers introducing non-pharmaceutical interventions in response to a rising mortality rate, which would downwards bias the effect of introducing the interventions. It also allowed us to examine effects of multiple policies concurrently, drawing on inter-country intervention variation. Evidence suggests a mean lag between virus transmission and symptom onset of 6 days [24, 25], and a further mean lag of 18 days between onset of symptoms and death [26], resulting in an overall likely mean lag of 24 days between intervention introduction and associated Covid-19 mortality. This duration underpins a choice of 24-day periods for modelling impacts and the decision to measure implementation timing and strictness prior to the period of mortality analysis.

We considered two distinct time periods of 24 days to account for the changing magnitude of effects of an exponentially transmitted virus: i) 0-24 days and ii) 14-38 after the first Covid-19 indexed death. The analysis at 0-24 days is very unlikely to suffer from bias caused by reverse causality since policymakers cannot possibly be reacting to deaths within their own country. The 14-38 days analysis, on the other hand, has a small risk of this bias if policymakers react quickly to deaths within the first 14 days. However, the mean number of cumulative deaths in the analysis sample at 14 days was 31 (SD 68) suggesting the mortality rate was still relatively low in most countries. Indexing all countries to these relative mortality dates also had the benefit of comparing country interventions at comparable stages of the pandemic.

For each of the two 24-day periods we calculated, and fixed for each country, the strictness and timing of each of the nine non-pharmaceutical interventions prior to the start of the period, i.e. strictness/timing of interventions implemented before the day of the first reported Covid-19 death were analysed for the 0-24 day period analysis; and strictness/timing of interventions up to 14 days after the first reported Covid-19 death were analysed for the 14-38 day period analysis.

#### Primary intervention measure

For our primary analysis we generated an aggregated measure of both strictness and timeliness to account for the changes in interventions over time, equivalent to an interaction of strictness and timing. Mean intervention strictness was calculated for each of the 24-day time periods: i) from 30 days before (to ensure comparable time periods over which the average was taken) the first Covid-19 death; and ii) from 30 days before until 14 days after the first Covid-19 death. Countries with earlier and stricter non-pharmaceutical interventions, therefore, had higher mean strictness values.

#### Secondary intervention measure

Due to the dynamic nature of policy implementation, it is inadequate to observe only intervention strictness or timing alone, which is why we prefer our primary analysis measure. However, additionally separating analysis by strictness or timing alone assisted in interpretation of the analysis of the combined mean score. For secondary analysis we took the maximum strictness value (see Table 1) for each intervention in each country over the analysis period. To account for differences in the start date of virus transmission in each country and ensure country comparability, we constructed a variable which counted the days between the implementation of an intervention and the first recorded Covid-19 death (e.g., − 10 if implemented 10 days before the first recorded Covid-19 death). Intervention timing was modelled categorically to avoid imposing linear effects.

For all analyses we ran multivariable regression models of the lagged (i.e., i) prior to first Covid-19 death; ii) 14-days-post first Covid-19 death) nine concurrent non-pharmaceutical intervention statuses on daily Covid-19 deaths per million population over the subsequent 24-day period. This means we did not overly-rely on the accuracy of the 24-day period informed by the literature, able to capture daily deaths at any point from 0 to 38 days over the combined analyses. We also included a range of covariates (see Table 2), a set of categorical indicators for day-of-the-week and a set of categorical indicators for week-of-the-year to capture seasonality, and a time fixed effect (number of days since first death in country) to account for the magnitude of effects of death varying over the 24-day analysis period due to exponential virus spread. Standard errors were clustered at the country-level. Further details on the empirical strategy are provided in the Additional file (section 1).

We excluded countries with less than a population of 100,000 and we analysed data for countries (*n* = 130) with no missing intervention indicators, outcome, or covariates at each time point.

### Robustness analysis

Firstly, there may be bias from varying definitions of a Covid-19 attributed death and recording practices across countries. The European Observatory categorises deaths as either ‘clinical-diagnosis-based’ or ‘test-based’ [28]. Country’s choice of definition may over/under-inflate reported Covid-19 deaths and may be associated with the timing/stringency of non-pharmaceutical interventions. To examine this, for the 28 countries with available data on death definition, we used logistic regressions to examine the association between each mean non-pharmaceutical intervention implementation and the Covid-19 death definition.

Secondly, accuracy of Covid-19 deaths may also vary due to challenges in determining underlying cause of death. For example, there have been questions over the accuracy of mortality reporting in China [29], and there was a high per-capita Covid-19 mortality rate in Belgium due to the inclusion of all deaths in care homes regardless of cause [30]. We therefore conducted a robustness analysis omitting these two countries from analyses.

Thirdly, as daily deaths per million is an over-dispersed count variable, we repeated all analyses using negative binomial regression (reporting incidence rate ratios) to check the consistency of the direction and significance of intervention effect across non-linear models.

## Results

**Fig. 1:**
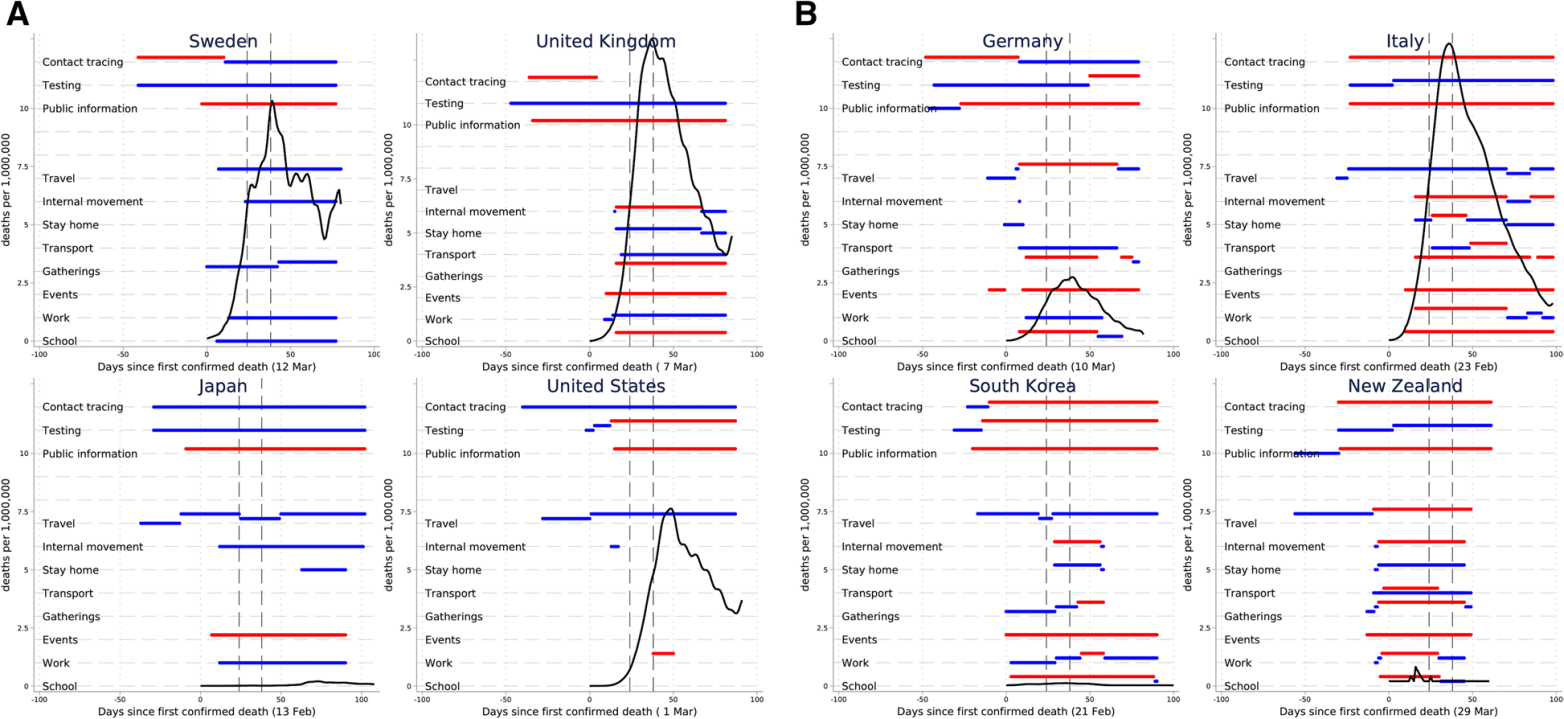
Implementation of non-pharmaceutical interventions and daily Covid-19 death rates for 8 selected countries. Notes: Horizontal line indicates strictness of implementation, with maximum implementation in red, any other implementation in blue. Locally weighted regressions (bandwidth = 0.2) of the raw daily deaths per million on time. Dashed vertical lines identify the periods of analysis, 24- and 38-days after first confirmed Covid-19 death. Date of first confirmed death observed in parenthesis

Figure 1 shows the introduction of non-pharmaceutical interventions to address Covid-19 transmission and daily Covid-19 deaths for selected countries (see Additional file section 2 for cumulative deaths for individual countries included in the analysis). Notably, elucidating mortality impacts from separate interventions using visual aids, or statistically without controlling for those co-introduced, is problematic given the introduction of multiple interventions.

In unadjusted analysis (illustrated in the scatter graphs in Additional file section 3), earlier and stricter intervention implementation of all nine non-pharmaceutical interventions were associated with lower Covid-19 deaths, as expected, although effects were small in magnitude. Table 3 shows the variation in intervention exposure across countries in each analysis period.

**Table 3:** Country variation in timing/strictness of policies at each analysis period

1. Policies introduced before (*N* = 130)			Mean (S.D.) / n(%)	2. Policies introduced up to 14 days post-first death (*N* = 126)			Mean (S.D.) / n(%)
Cumulative deaths (24 days)			182.4 (600.9)	Cumulative deaths (38 days)			760.6 (2499.9)
Cumulative deaths (24 days, per million population)			8.4 (17.6)	Cumulative deaths (38 days, per million population)			24.3 (62.1)
**School (strictness)**		**School (timeliness)**		**School (strictness)**		**School (timeliness)**	
Not introduced	36 (27.7%)	Not introduced	36 (27.7%)	Not introduced	12 (9.5%)	Not introduced	12 (9.5%)
Least strict	1 (0.8%)	0-10 days before first death	43 (33.1%)	Least strict	1 (0.8%)	Introduced before first death	90 (71.4%)
2	8 (6.2%)	11-20 days before first death	41 (31.5%)	2	5 (4.0%)	0-7 days after first death	17 (13.5%)
3	85 (65.4%)	21+ days before first death	10 (7.7%)	3	108 (85.7%)	8-14 days after first death	7 (5.6%)
**Work (strictness)**		**Work (timeliness)**		**Work (strictness)**		**Work (timeliness)**	
Not introduced	65 (50.0%)	Not introduced	65 (50.0%)	Not introduced	29 (23.0%)	Not introduced	29 (23.0%)
Least strict	14 (10.8%)	0-10 days before first death	43 (33.1%)	Least strict	12 (9.5%)	Introduced before first death	62 (49.2%)
2	25 (19.2%)	11-20 days before first death	17 (13.1%)	2	28 (22.2%)	0-7 days after first death	27 (21.4%)
3	26 (20.0%)	21+ days before first death	5 (3.8%)	3	57 (45.2%)	8-14 days after first death	8 (6.3%)
**Events (strictness)**		**Events (timeliness)**		**Events (strictness)**		**Events (timeliness)**	
Not introduced	34 (26.2%)	Not introduced	34 (26.2%)	Not introduced	14 (11.1%)	Not introduced	14 (11.1%)
Least strict	10 (7.7%)	0-10 days before first death	46 (35.4%)	Least strict	5 (4.0%)	Introduced before first death	92 (73.0%)
2	86 (66.2%)	11-20 days before first death	37 (28.5%)	2	107 (84.9%)	0-7 days after first death	15 (11.9%)
		21+ days before first death	13 (10.0%)			8-14 days after first death	5 (4.0%)
**Gatherings (strictness)**		**Gatherings (timeliness)**		**Gatherings (strictness)**		**Gatherings (timeliness)**	
Not introduced	52 (40.0%)	Not introduced	52 (40.0%)	Not introduced	25 (19.8%)	Not introduced	25 (19.8%)
Least strict	4 (3.1%)	0-10 days before first death	40 (30.8%)	Least strict	4 (3.2%)	Introduced before first death	76 (60.3%)
2	7 (5.4%)	11-20 days before first death	30 (23.1%)	2	5 (4.0%)	0-7 days after first death	21 (16.7%)
3	34 (26.2%)	21+ days before first death	8 (6.2%)	3	33 (26.2%)	8-14 days after first death	4 (3.2%)
4	33 (25.4%)			4	59 (46.8%)		
**Transport (strictness)**		**Transport (timeliness)**		**Transport (strictness)**		**Transport (timeliness)**	
Not introduced	92 (70.8%)	Not introduced	92 (70.8%)	Not introduced	65 (51.6%)	Not introduced	65 (51.6%)
Least strict	18 (13.8%)	0-10 days before first death	25 (19.2%)	Least strict	32 (25.4%)	Introduced before first death	36 (28.6%)
2	20 (15.4%)	11-20 days before first death	9 (6.9%)	2	29 (23.0%)	0-7 days after first death	20 (15.9%)
		21+ days before first death	4 (3.1%)			8-14 days after first death	5 (4.0%)
**Stay home (strictness)**		**Stay home (timeliness)**		**Stay home (strictness)**		**Stay home (timeliness)**	
Not introduced	81 (62.3%)	Not introduced	81 (62.3%)	Not introduced	37 (29.4%)	Not introduced	37 (29.4%)
Least strict	17 (13.1%)	0-10 days before first death	30 (23.1%)	Least strict	25 (19.8%)	Introduced before first death	48 (38.1%)
2	20 (15.4%)	11-20 days before first death	16 (12.3%)	2	49 (38.9%)	0-7 days after first death	27 (21.4%)
3	12 (9.2%)	21+ days before first death	3 (2.3%)	3	15 (11.9%)	8-14 days after first death	14 (11.1%)
**Internal movement (strictness)**		**Internal movement (timeliness)**		**Internal movement (strictness)**		**Internal movement (timeliness)**	
Not introduced	79 (60.8%)	Not introduced	79 (60.8%)	Not introduced	38 (30.2%)	Not introduced	38 (30.2%)
Least strict	17 (13.1%)	0-10 days before first death	33 (25.4%)	Least strict	25 (19.8%)	Introduced before first death	49 (38.9%)
2	34 (26.2%)	11-20 days before first death	15 (11.5%)	2	63 (50.0%)	0-7 days after first death	21 (16.7%)
		21+ days before first death	3 (2.3%)			8-14 days after first death	18 (14.3%)
**Travel (strictness)**		**Travel (timeliness)**		**Travel (strictness)**		**Travel (timeliness)**	
Not introduced	16 (12.3%)	Not introduced	16 (12.3%)	Not introduced	5 (4.0%)	Not introduced	5 (4.0%)
Least strict	13 (10.0%)	0-10 days before first death	23 (17.7%)	Least strict	1 (0.8%)	Introduced before first death	110 (87.3%)
2	9 (6.9%)	11-20 days before first death	22 (16.9%)	2	5 (4.0%)	0-7 days after first death	9 (7.1%)
3	33 (25.4%)	21+ days before first death	69 (53.1%)	3	33 (26.2%)	8-14 days after first death	2 (1.6%)
4	59 (45.4%)			4	82 (65.1%)		
**Public information (strictness)**		**Public information (timeliness)**		**Public information (strictness)**		**Public information (timeliness)**	
Not introduced	12 (9.2%)	Not introduced	12 (9.2%)	Not introduced	3 (2.4%)	Not introduced	3 (2.4%)
Least strict	10 (7.7%)	0-10 days before first death	12 (9.2%)	Least strict	6 (4.8%)	Introduced before first death	114 (90.5%)
2	108 (83.1%)	11-20 days before first death	26 (20.0%)	2	117 (92.9%)	0-7 days after first death	6 (4.8%)
		21+ days before first death	80 (61.5%)			8-14 days after first death	3 (2.4%)
**Testing (strictness)**		**Testing (timeliness)**		**Testing (strictness)**		**Testing (timeliness)**	
Not introduced	19 (14.6%)	Not introduced	19 (14.6%)	Not introduced	11 (8.7%)	Not introduced	11 (8.7%)
Least strict	76 (58.5%)	0-10 days before first death	18 (13.8%)	Least strict	61 (48.4%)	Introduced before first death	108 (85.7%)
2	30 (23.1%)	11-20 days before first death	27 (20.8%)	2	43 (34.1%)	0-7 days after first death	3 (2.4%)
3	5 (3.8%)	21+ days before first death	66 (50.8%)	3	11 (8.7%)	8-14 days after first death	4 (3.2%)
**Contact tracing (strictness)**		**Contact tracing (timeliness)**		**Contact tracing (strictness)**		**Contact tracing (timeliness)**	
Not introduced	29 (22.3%)	Not introduced	29 (22.3%)	Not introduced	17 (13.5%)	Not introduced	17 (13.5%)
Least strict	44 (33.8%)	0-10 days before first death	21 (16.2%)	Least strict	48 (38.1%)	Introduced before first death	98 (77.8%)
2	57 (43.8%)	11-20 days before first death	29 (22.3%)	2	61 (48.4%)	0-7 days after first death	8 (6.3%)
		21+ days before first death	51 (39.2%)			8-14 days after first death	3 (2.4%)

Mean strictness/timing of non-pharmaceutical interventions were not associated with Covid-19 death definitions suggesting death definition is unlikely to bias the estimated coefficients (see Additional file section 4).

### Primary analysis

**Fig. 2:**
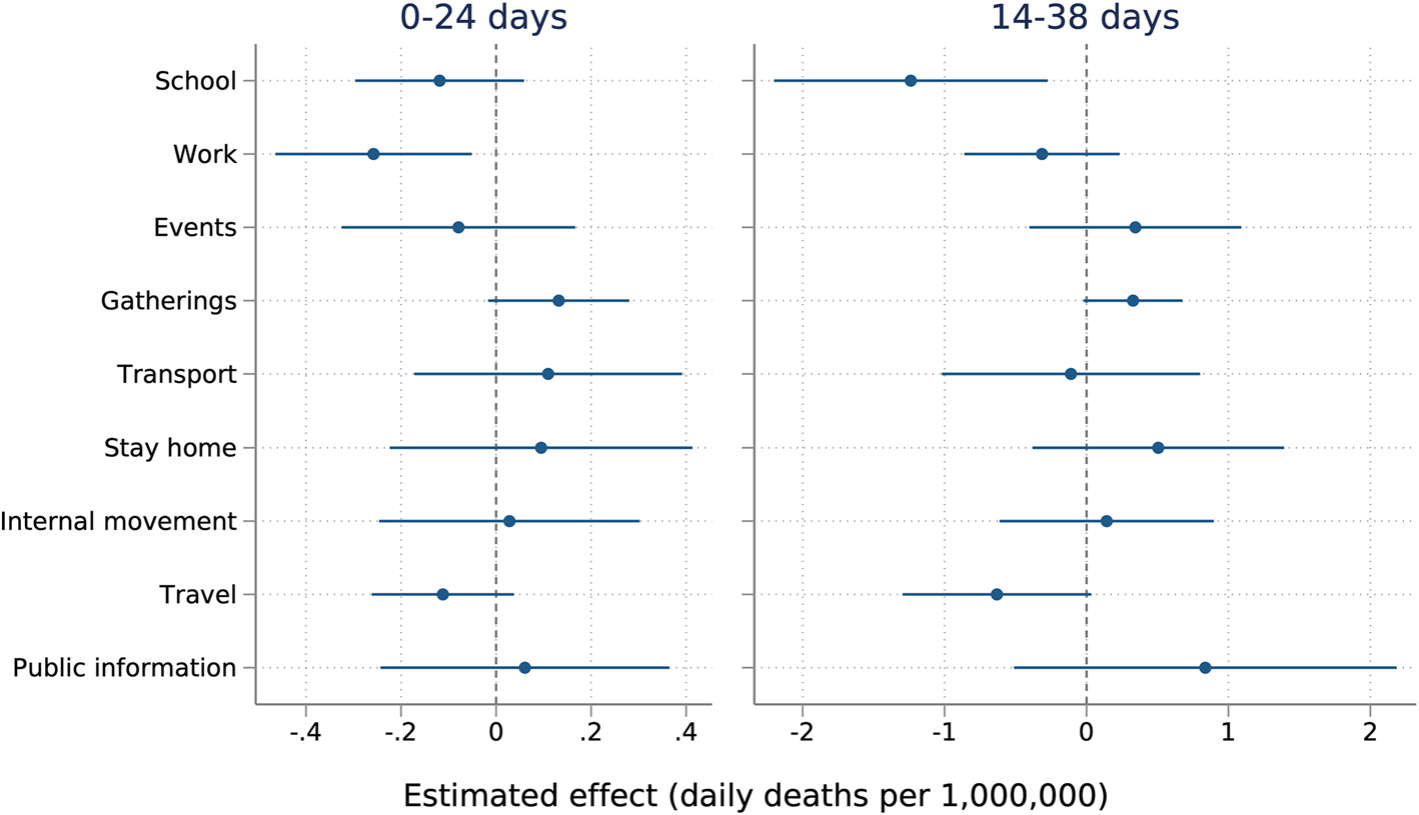
Regression results examining intervention strictness and timing combined (mean score). Notes: Estimated parameters of two regressions adjusted for a range of covariates (Table 2), a set of categorical indicators for day-of-the-week and a set of categorical indicators for week-of-the-year to capture seasonality, and the time (number of days since first death in country) to account for the magnitude of effects of death varying over the 24-day analysis period due to exponential virus spread. Standard errors were clustered at the country-level. Sample size: 130 countries (3250 observations) for 0-24 days analysis; 126 countries (3150 observations) for 14-38 days analysis

Figure 2 shows the results from the pooled cross-sectional regression analysis including both intervention strictness and timing (through the mean score). Stricter/earlier workplace closures were associated with fewer Covid-19 deaths in the early part of the epidemic (first 24 days), − 0.26 per million (95% CI -0.46 -0.05) with a one unit increase in the mean score (equivalent to, for example, a strictness score of 1 over the entire time period, 2 for half the time period, 3 for a third of the time period, or 4 for a quarter of the time period). In the 14-38 days analysis, where the magnitudes are expected to be larger due to exponential virus spread, stricter/earlier school closures were associated with the largest reductions in Covid-19 deaths of − 1.23 per million (95% CI -2.20 -0.27). Mean scores of other interventions were not associated with Covid-19 mortality.

Early/strict school closing was consistently associated with lower mortality rates across all robustness checks in the 14-38 days analysis and had a negative but not statistically significant estimate in all 0-24 days analyses (see Additional file section 5). Stricter/earlier workplace closures was robust to the analysis without China/Belgium at 0-24 days. The result was negative but not significant in the negative binomial model. The estimate was also statistically significant in the 14-38 days analysis without China/Belgium. Earlier/stricter restrictions on gatherings was not significant in the primary analysis but was significantly associated with a slightly larger mortality rate in both robustness checks at 0-24 days and only in the analysis without China/Belgium at 14-38 days.

### Robustness analysis

Results from the fully-adjusted pooled cross-sectional regressions examining intervention strictness alone and timing alone (see Additional file section 5) show that implementation of school closing prior to first recorded death appears to be driving the effect of school closing on mortality rates, with required rather than recommended closing (least strict) also potentially preferable. The driver of the workplace finding was less obvious, but again the earlier implementation appeared to be the main driver of the result.

Results from additional robustness analyses (see Additional file section 5) included stricter international travel controls consistently associated with lower Covid-19 mortality rates in the 14-38 days analyses. Strictness of restrictions on gatherings was associated with slightly lower Covid-19 mortality rates in the 14-38 days analyses. Later restrictions on gatherings were also associated with lower Covid-19 mortality across all 14-38 analyses. Later implementers tended to introduce the strictest version (restrictions on gatherings of less than 10 people) compared to earlier implementers of this intervention (75% implementing the strictest version 8-14 days after first death, versus 50% at 0-7 days and 60% of those that introduced before first death).

## Discussion

Using data from 130 countries, we examined the effects of non-pharmaceutical interventions on Covid-19 attributed mortality. We fixed information on intervention implementation prior to the period over which we analysed deaths to mitigate the bias from reverse causality. We found that earlier and stricter implementation of some non-pharmaceutical interventions contributed to relatively lower numbers of Covid-19 deaths. Notably, the earlier/stricter implementation of school and workplace closures were associated with lower Covid-19 mortality rates. Without controlling for intervention timing stricter international travel controls, and without controlling for intervention strictness later restrictions on gatherings, were also associated with lower Covid-19 mortality across all 14-38 day analyses.

There are key limitations of this analysis to consider when interpreting the findings. First, although we control for a range of potential confounders, these were proxies where data was available across countries and do not necessarily capture all important variation. Other potential confounders might be available, for instance proxies like labour force participation rate, and numbers of formal workers to proxy for the proportion of the population who were ‘essential workers’ [31]. However, these measures would likely be highly correlated with confounders already included in our models, such as GDP. There is still, therefore, a risk of unobserved time-varying confounding. We were not able to add country fixed-effects because we fixed the intervention profile in each country for the analysis period to address reverse causality concerns. Instead of within-country variation (as in fixed-effects), we relied on available cross-country policy variation and co-variates to adjust for important between-country differences. Other methods typically used to examine causal effects of interventions, such as difference-in-differences, are biased in settings with multiple policies implemented across time and geography, because pre-intervention trends for one intervention are impacted by any effects of pre-existing policies [32]. This lack of available methods is more generally applicable to any analysis of Covid-19 policies, to date there is no best practice for evaluation of separate policies in the context of multiple co-introduced strategies. Instead, we use the known biological lag period between intervention effect on transmission and our outcome of interest and adjusted for multiple policies simultaneously to produce as robust a result as possible.

Secondly, this study only examines the impact of nationally recorded policies using national aggregate data, meaning subnational interventions were not captured. Furthermore, we were unable to measure compliance and regional variation in implementation, as well as voluntary changes in population behaviours or effects of individual-level covariates. We were also unable to estimate longer-term effects due to limited statistical power and increased risk of bias due to reverse causality in later periods. A key limitation is the nature of these interventions. Whilst we examine them independently and look for separate effects, there are likely to be interaction effects. Additionally, we examine a narrow window of time in the initial stages of wave one of the outbreak. Alternative analysis methods will likely be necessary to reduce bias of reverse causality in later periods, however, as policymakers are likely to have implemented stricter policies in response to higher mortality rates.

Thirdly, we were only able to examine a single, albeit important, outcome, mortality, due to comparable data availability and the necessity of a known lag period between intervention and outcome. It is not possible to estimate effects on transmission or mobility in this way, for example, as the interventions should directly and immediately affect these outcomes with no lag to exploit. The relative effectiveness of non-pharmaceutical interventions against other outcomes, such as mental health and economic outcomes, and potential trade-offs across outcomes, are also important. As noted above, there is also variation in definitions and accuracy of mortality reporting across countries [28], however robustness checks and adjusting for testing and contact tracing policies suggest this does not greatly impact the findings. Excess mortality, all-cause mortality compared to previous years, is arguably a better measure to also take account of unattributed and indirect deaths, as well as more accurately capturing death from- rather than with-Covid-19 early in the pandemic. Unfortunately, excess mortality relies on calculations (compared to a pre-period) and is not comparatively available for as many countries, necessary for statistical power to incorporate concurrent interventions. However, a recent study also showed that the mortality data we use is able to correctly signal the true trajectory of the trend when compared to excess mortality data available in 17 countries [33]. Mortality also lies on a complex pathway from initial virus transmission that involves population health and behaviours, underlying health inequalities, and healthcare access and quality. Further understanding the patterns and causal mechanisms which have affected mortality rates is essential for future outbreaks but is not possible with current data.

The findings from this study align with evidence from previous epidemics that also concluded that interventions such as workplace closures, school closures, and quarantine periods were most effective for reducing cases [13]. There was previously much less evidence available on the effects of internal and international travel restrictions, bans on mass gatherings and public information campaigns [13]. A rapid systematic review early in the Covid-19 pandemic examining the effects of school closures reported a dearth of evidence on the topic, but highlighted that modelling studies assumed a 2-4% reduction in deaths, much less than attributed to other non-pharmaceutical interventions [14].

Early published Covid-19 specific studies on the impacts of single policies mostly focused on stay-at-home interventions. For example, a Californian study estimated 1661 fewer deaths over 1 month from the introduction of a stay-at-home intervention (at an estimated cost of 400 job losses per life saved) [15], whilst a study from US states estimated stay-at-home interventions were associated with approximately 50% fewer deaths, with early adopting states experiencing larger reductions in mortality [16]. The impact of stay-at-home interventions were also contrasted between Denmark and Sweden which suggested the number of deaths would have been 167% higher in Denmark without stay-at-home interventions [17]. These findings contrast to our study which found no association between mandatory stay-at-home interventions on cross-country Covid-19 mortality after adjusting for other non-pharmaceutical interventions concurrently introduced. One study examining the effects of multiple policies in Hong Kong shows that the combined package of policies introduced, including border restrictions, quarantine, and social distancing, were associated with reduced transmission of Covid-19 [18]. A recent study also showed that school closures, particularly implemented early, have been effective at reducing incidence (− 62%) and mortality (− 58%) in the US [20].

Other cross-country comparisons of non-pharmaceutical interventions have identified that travel restrictions were associated with slower geographical spread and initial case numbers, but failed to quantify the specific effect [19]. One study from six countries (China, South Korea, Italy, Iran, France, and the US) predicted that combined non-pharmaceutical interventions might have prevented or delayed a total of 62 million cases [2]. Our findings are also consistent with a study examining interventions implemented in 30 European countries which reported school closures, non-essential business closures, and prohibiting mass gatherings to be effective in reducing Covid-19 deaths [34]. A large cross-country comparison examined effects of multiple interventions simultaneously on disease incidence, combining interrupted time series and meta-analysis. The authors reported a general overall finding that “physical distancing interventions were associated with reductions in the incidence of covid-19 globally” [1], but were not able to look at relative effects. Nevertheless, detailed examination of their findings shows that the largest effect on reducing incidence was a combination of school closures, workplace closures and restrictions on mass gatherings, directly in line with our findings.

Our results are most consistent with those using statistical methods to examine relative effects on the effective reproduction number of Covid-19. Haug et al. likewise found that small gathering cancellations (which included mandatory home working in their case) and closure of education facilities were the most effective interventions. Although they found that stay at home interventions could also reduce transmission, they concluded that “less disruptive and costly NPIs [interventions] can be as effective as more intrusive, drastic, ones (for example, a national lockdown)” [22]. Liu et al., similarly incorporating 130 countries, found a strong association for two interventions (school closures and internal movement restrictions), and reduced reproduction number also for another three (workplace closure, income support, and debt/contract relief). Public events cancellation and restrictions on gathering only had strong evidence at their maximum strictness level. Evidence for other interventions affecting reproduction number, including stay-at-home requirements, “was inconsistent and inconclusive” [23].

### Implications for policy and practice

The finding that selected non-pharmaceutical interventions are associated with lower Covid-19 mortality is not surprising given the known reductions in social contacts and transmission from these interventions, and their historical use to control epidemics. However, it may be unexpected that workplace and, particularly, school closures were associated with relatively lower Covid-19 mortality across countries whilst interventions such as stay-at-home measures were not. One plausible interpretation is that schools and workplaces involve ‘compulsory’ interactions with others, as individuals feel obliged to attend in person and may be concerned for loss of earning or educational opportunities. This compares to interventions targeting other sources of human interaction which are more ‘voluntary’ and may reduce irrespective of whether mandated policies are introduced (therefore giving no additional observable effect of introducing the intervention).

This mechanism is supported by multiple studies examining the effects of non-pharmaceutical interventions on mobility. One US study found mobility reductions in all US states, even those not adopting formal stay-at-home measures, due to individuals’ voluntary behaviour changes [35]. Notably, mobility fell *prior* (i.e. could not have been an effect of the intervention, as effect must follow cause) to stay-at-home implementation in US states which introduced measures [36], and mobility fell a comparable amount (52%) in US counties *without* stay-at-home measures compared to those with (61%) [37]. Likewise, examination of Google mobility data in Sweden, with a lack of stay-at-home measures, shows mobility nevertheless decreased in retail and workplaces by roughly − 25% and transit − 35% by end of March [38]. Unfortunately, this mobility data is not comparable across countries in order to analyse this specific mechanism within this study. Furthermore, significant voluntary response, separate from direct policy response, has also been evidenced following locally/nationally reported Covid-19 case counts [39, 40]. This voluntary response may be driven by risk perception of the population [41]. We do not suggest that these other interventions are not effective at reducing social contacts, therefore, but the cumulative evidence above suggests that mandatory introduction might be unnecessary if people change their social interaction behaviours voluntarily.

School closing having the largest effect is also interesting, in that children are known to be relatively less personally affected by the negative effects of Covid-19 than older adults [42]. This result highlights the well-documented problem of asymptomatic transmission in Covid-19 [43]. The extremely low individual risk to these groups might encourage more risky behaviour which can subsequently affect wider households and communities. This relative risk behaviour was seen in media around the world reporting house parties/raves and u-turns on policies opening University campuses as infection rates subsequently accelerated upon re-opening, for instance [44, 45]. There is the need to examine specific interventions to incentivise societally-optimal behaviour and internalising of the population-level risk for these low-risk individuals. Instead of closing, there might also be less harmful interventions, such as daily symptoms screens, effective masking, that could be implemented in ‘compulsory’ settings such as schools/workplaces as a more nuanced policy approach in the future [46].

Overall, policymakers should note that efforts to reduce social interactions through restricting compulsory activities, such as school and work, may be more effective in reducing mortality than targeting actions where individuals have more freedom in choice or where activities are likely diminished due to other actions. However, our results only apply directly to the early stages, wave one, of the Covid-19 pandemic. For identifying responses to subsequent waves, or other novel pathogens, understanding the complex behaviour changes from specific interventions is necessary for appropriate mitigation. There is no guarantee that the behaviour changes outlined above would be replicated if population complacency or a primary focus on other immediate pressures, such as financial hardship, prevailed.

### Future research

Future research should examine long-term effects of interventions, such as whether countries suppressing stricter/earlier are more vulnerable to future outbreaks. Understanding the inter-connected nature of different interventions and their impacts on population behaviours, including how these behaviours change over time, is key. The important role of different countries’ health systems, supply chains and/or cultures, and ‘pre-existing conditions’ (in terms of population health, but also health of the systems and their ability to respond to ‘shocks’) should also be understood as important mediators in preventing Covid-19 mortality. Understanding how the targeting of specific interventions to certain populations and protecting high-risk groups (e.g. elderly) and institutions (e.g. hospitals and care homes) will hopefully reveal more nuanced policy responses for future outbreaks. Integrating policy options into wider behavioural science, for example through nudging or encouraging select behaviours [47], might offer long-term solutions that are more acceptable to populations. Lastly, future research should quantify the trade-offs against the large economic considerations that not only have monetary costs, but profound societal and long-term health impacts [4, 5]. Addressing methodological gaps to evaluate multiple policies is likely to be a key requirement for future health policy research. In the shorter-term, future systematic review and meta-analysis of multiple publications using currently available (each individually flawed) methods might be a means of increasing certainty.

## Conclusions

Early workplace and, particularly, school closures were associated with the lowest Covid-19 wave one mortality rates across 130 countries. Focusing on protecting individuals from social interactions by targeting more ‘compulsory’ places (including schools and workplaces) as opposed to more ‘voluntary’ interactions and changing behaviours of those with lower individual-risk appear to have been most effective strategies mitigating early Covid-19 mortality.

## Supplementary Information

**Additional file 1.**

## Data Availability

The datasets analysed during the current study are available from: -Intervention data: https://www.bsg.ox.ac.uk/research/research-projects/covid-19-government-response-tracker -Covid-19 mortality data: https://www.ecdc.europa.eu/en/geographical-distribution-2019-ncov-cases
